# Short-Term Effect of Ankle Eversion Taping on Bilateral Acute Ankle Inversion Sprains in an Amateur College Football Goalkeeper: A Case Report

**DOI:** 10.3390/healthcare8040403

**Published:** 2020-10-15

**Authors:** Jung-Hoon Lee

**Affiliations:** 1 Department of Physical Therapy, College of Nursing, Healthcare Sciences and Human Ecology, Dong-Eui University, Busan 47340, Korea; dreampt@hanmail.net; Tel.: +82-51-890-4222; 2 Integrated Physical Medicine Institute, Dong-Eui University, Busan 47340, Korea

**Keywords:** ankle, ankle inversion sprain, kinesiology tape, football

## Abstract

This case study aimed to investigate the short-term effects of ankle eversion taping (AET) using kinesiology tape on bilateral acute ankle inversion sprains in an amateur college soccer goalkeeper. Ankle eversion taping was applied for two weeks (average 16 h/day) on a 24-year-old goalkeeper with bilateral grade 2 acute ankle inversion sprain with swelling (left ankle more severe) during a soccer match. The subject had a foot ankle outcome score (FAOS) of 41%; visual analog scale (VAS) scores of 5/10 and 7/10 for the right and left ankles, respectively; patient-specific functional and pain scale (PSFS) score of 12/50; and limited range of motion of the ankle. The swelling disappeared after AET in both ankles. In the weight-bearing lunge test, the right and left ankle distances increased from 2 cm to 12 cm, and from 0 cm to 12 cm, respectively. The FAOS improved from 20% to 97%, while the PSFS score improved from 12/50 to 50/50. The VAS scores decreased to 0/10 for both ankles. AET is a potential clinical treatment method for acute ankle inversion sprain with swelling.

## 1. Introduction

Soccer, which involves complex movements such as running, jumping, landing and rapid turnover, has a high risk of damage [1]. The incidence of ankle injury is the highest [2], and approximately 80% of all ankle sprains are associated with the lateral ligament complex [3]. After spraining their ankle, more than 40% of patients continue to experience ankle instability or wobbling despite no structural restrictions [4] and develop chronic ankle instability [5]. In soccer players, loss of muscle strength and balance caused by ankle instability leads to increased instability, recurrent ankle damage, and decreased performance [6]. 

The common treatment for acute ankle sprains is protection, rest, ice, compression, and elevation (PRICE) [7,8]. Up to one-third of lateral ankle sprains can result in chronic damage due to continuous swelling, pain, and activity limitation [9]. Ankle braces are effectively used to prevent recurrence of ankle sprains [10,11]. However, some recent studies suggest that early movement accelerates recovery compared to rest [12,13]. Benefits of early movement for acute injuries include reduced swelling and pain, reduced stiffness, and early return to work by preserving joint motion [12].

Kinesiology taping has been used to prevent ankle sprain injury in fatigued ankles [14], improve ankle balance [15], and recover chronic ankle instability [16]. However, no research has been conducted on acute ankle inversion sprain recovery in goalkeepers versus other players. In addition, urgent intervention is required for a goalkeeper with an acute inversion sprain and swelling in both ankles. Therefore, we aimed to investigate the short-term effects of ankle eversion taping (AET) using kinesiology tape on bilateral acute ankle inversion sprains in amateur college soccer goalkeeper.

## 2. Case Report

A 24-year-old amateur college goalkeeper (height 176 cm; mass 85 kg; BMI 27.44) with no history of ankle sprain had experienced an acute inversion sprain of the right ankle while blocking a ball kicked by an opponent during a soccer match. For the next 30 min of the game, the subject endured the pain without requesting a substitute. In the process of defending the ball again, the subject experienced an acute ankle inversion sprain of the left ankle and was then substituted.

He was diagnosed with a grade 2 acute inversion sprain of both ankles by a medical physician. The evaluation was conducted before the first AET and after the final AET, except for swelling evaluation, by the same physical therapist with more than 10 years of experience. A swelling evaluation was performed daily. In the initial evaluation, swelling of the bilateral ankles was noted using the figure-of-eight-20 method [17], with a high interest of 0.99 and intra-test reliability. The results for the left and right ankles were 54 cm/53 cm and 57 cm/53 cm, respectively. On the visual analog scale (VAS) (0, no pain; 10, the worst imaginable pain), the left and right ankles scored 5/10 and 7/10, respectively. In the weight-bearing lunge test, which is often used to measure ankle dorsiflexion range of motion deficits in subjects with clinical ankle instability [18], the distance from the wall to the first toe was 2 cm and 0 cm in the right and left ankles, respectively (Table 1). The score of the patient-specific functional and pain scale (PSFS), an evaluation tool for functional status (0, unable to perform the activity; 10, able to perform the activity) [19], was 17/50 (Table 2). The foot ankle outcome score (FAOS), which was developed to allow patients to evaluate various foot- and ankle-related problems, consists of five subscales: pain, symptoms, activities of daily living, function in sports and recreation, and foot- and ankle-related quality of life [20]. The subject’s FAOS was 41%. 

**Figure 1:**
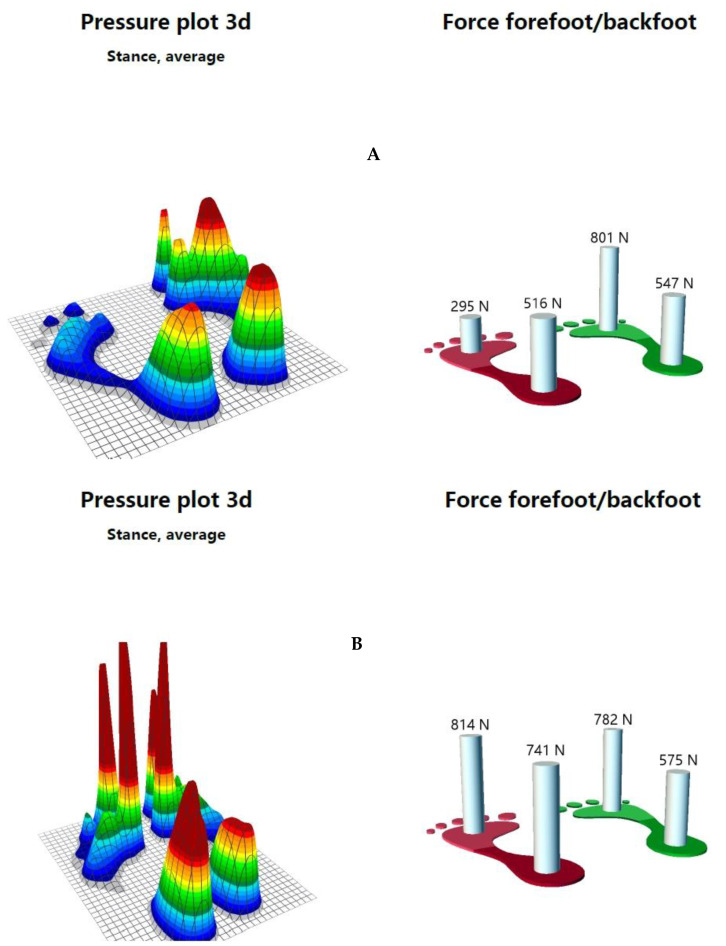
Foot pressure during walking ((**A**), before ankle eversion taping; (**B**), after ankle eversion taping).

Using Zebris (PDM-SX, zebris Medical GmbH, Isny, Germany), a device that electronically records and analyzes static balance and foot pressure, the static balance for duration of 1 min and 30 s (Table 3) and average pressure distribution of the soles using the mid-gait method during walking were measured (Figure 1A). Center of pressure (COP) path length (mm) was the overall length of COP path movement for a certain period [21,22]. In a previous study, the reduction in COP path length indicated an improvement in balance ability [23]. Written informed consent was obtained from the subject prior to the performance and publication of this case report. 

**Figure 2:**
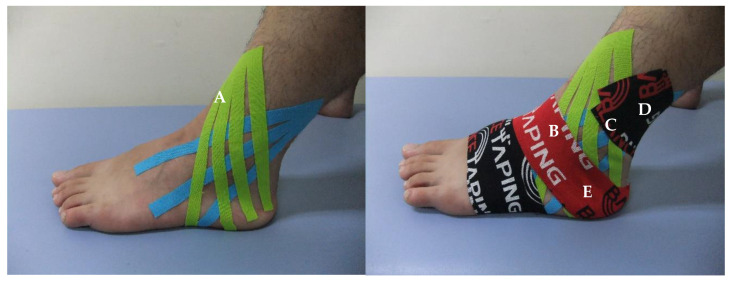
Ankle eversion taping using kinesiology tape for acute ankle inversion sprain ((**A**), fan-shaped strips of kinesiology tapes were applied to the lateral malleolus and dorsum area; (**B**), taping was applied from the talus to the calcaneus on both sides; (**C**), taping was applied to the medial calcaneus under the subtalar joint starting at 5 cm above the lateral malleolus and facing the medial side of the instep; (**D**), taping was applied in the same manner to overlap approximately 80% of the tape applied in the previous step; (**E**)**,** taping was applied over that from the first step).

To reduce swelling and pain caused by acute bilateral ankle inversion sprains, AET using kinesiology tape (BB TAPE, WETAPE Inc., Pyeongtaek, Korea) was applied to both ankles for two weeks (average 16 h/day) at approximately 30%–40% stretching by a physical therapist with over 10 years of taping treatment experience. Other interventions such as the PRICE method, physiotherapy, any kind of non-steroidal anti-inflammatory drug (NSAID), and pain-free exercise besides AET were not applied to the subject. Sports activities, including soccer games, were not performed. The daily activities that the subject was able to perform, were not limited during the application period of AET. To help reduce bilateral swelling before the use of AET, fan-shaped strips of kinesiology tapes (three fan tails to the right, and four fan tails to the left) were applied to the lateral malleolus and dorsum area, with approximately 10% stretch (Figure 2A).

The AET method consists of four steps [24,25]. First, for the posterior talar glide to increase ankle dorsiflexion, the ankle was slightly dorsiflexed and the tape was applied from the talus to the calcaneus on both sides (Figure 2B). Second, to induce ankle eversion, the ankle was everted until pain was induced, and the tape was applied to the medial calcaneus under the subtalar joint starting at 5 cm above the lateral malleolus and facing the medial side of the instep (Figure 2C). Third, to strengthen the mechanical correction effect to maintain ankle eversion, the kinesiology tape was applied in the same manner to overlap approximately 80% of the tape applied in the previous step (Figure 2D). Finally, to strengthen the posterior talar glide and increase ankle dorsiflexion, while the ankles were held in a slight dorsiflexion state, the tape was applied over that from the first step (Figure 2E). To prevent skin irritation, the start and end points of the I-shaped elastic tape (approximately 2 cm) were applied without stretching [24,26], and the applied AET was removed within 24 h and reapplied daily, even if the subject did not experience itching [24,26].

Evaluation of swelling using the figure-of-eight-20 method showed that the swelling of the right ankle decreased from 54 cm/53 cm to 53 cm/53 cm after two days and that of the left ankle decreased from 57 cm/53 cm to 53 cm/53 cm after one week, returning to normal. In the weight-bearing lunge test, the distance of the right ankle increased from 2 cm/12 cm to 12 cm/12 cm, while that of the left ankle increased from 0 cm/12 cm to 12 cm/12 cm (Table 1). The total PSFS score increased from 12/50 to 50/50 (Table 2), and in the VAS, both ankles scored 0/10 (Table 1). The FAOS increased from 41% to 97%, while the static balance ability and the average pressure distribution of the soles in walking stance also increased, as evaluated using Zebris (Figure 1B, Table 3). The subject had no complaints of pain during the standing squat position, heel lifting, or running. Thus, the subject was able to play again as a goalkeeper in soccer matches.

## 3. Discussion

In this case study, repeated AET reduced pain and swelling and increased self-reported functional ankle and range of motion in a case of bilateral acute ankle inversion sprain with swelling. The first treatment strategy for AET is to protect the sprained ankle from further injury and avoid inversion, the cause of the pain, through the mechanical effects of eversion. The elasticity of the kinesiology tape supports the ankle joint without limiting its range of motion while improving its movement [15]. Thus, when the ankle joint moves in everyday life, the kinesiology tape applied to the peripheral skin of the ankle stretches. As the tension of the tape increases, the elastic force aiming to return it to its original length restores the ankle joint to its normal position and accelerates the recovery process [16]. By applying AET with kinesiology tape, the more everted ankle would avoid inversion and dorsiflexion that caused pain in the sprained ankle. Therefore, the use of AET for two weeks would have helped in the natural healing process. The plantar pressure of the first evaluation seemed to protect the left foot (which had more acute sprain) by shifting body weight more to the right leg, and the heels achieved more equal pressure distribution due to pain reduction after AET application.

Proprioception detects the locations of the joints and all extremities after movement and helps them recover their normal positions [27]. Previous studies showed that the application of kinesiology tape enhanced proprioception by stimulating the cutaneous mechanoreceptors [28,29]. After the application of the kinesiology tape, with 30%–40% elasticity to the skin around an ankle with an inversion sprain, the skin area with the kinesiology tape is modified according to the ankle joint’s movements in daily life [30]. This stimulates cutaneous mechanoreceptors [28,31] and activates the ankle joint proprioceptors [28,32]. Therefore, the application of kinesiology tape to the ankle joint with an inversion sprain improves the self-reported functional ankle score of the FAOS and PSFS scores. Furthermore, it may have enhanced the plantar pressure by improving the sensed position of the ankle joint that supports static posture and walking using the Zebris to help maintain a normal position of the ankle joint. 

Although evidence supporting the effectiveness of kinesiology tape in decreasing swelling is lacking in the literature, in this case, the swelling disappeared after two days and one week in the right and left ankles, respectively. In a previous study, in patients with degree 1 and 2 ankle sprains, heat and contrast bath therapy increased the amount of ankle swelling almost equally three days after ankle injury, but cold therapy minimized swelling during three, four, and five days after ankle injury [33]. The application of kinesiology tape is thought to reduce swelling by stimulating the drainage of edema present in the interstitial space to less congested lymphatic channels. A previous study also showed reduced swelling after the kinesiology taping of fan-shaped strips for swelling after surgical correction of zygomatico-orbital fractures [34]. In contrast, in a case of acute ankle sprain similar to that described here, no clinically significant reduction in swelling was observed after the application of fan-shaped kinesiology tape strips [35]. In that study, certain limitations were observed. Fan-shaped kinesiology tape strips were not directly applied to the swelling area, and the tape was only applied for three days. In the current study, fan-shaped strips and AET were applied to the left and right ankles for one week and two days, respectively, with overlapping parts. Thus, the kinesiology tape was stretched to 30%–40% during application, which would have clinically mimicked the application of compression bandages to the swelling area. Thus, the continuous pressure would have reduced the swelling.

The current study has certain limitations. First, this study involved a single case; thus, the treatment method could not be compared with other methods. Second, more subjects could not be recruited at this time because acute ankle sprain with bilateral ankle swelling rarely occurs, especially when blocking the ball that was kicked by an opponent during a soccer game. Third, since the fan-shaped strips and AET were applied simultaneously, a comparative analysis of the effect of each method for reducing swelling could not be performed. In the future, it will be necessary to compare AET to ankle brace or sport tape using non-elastic tape and other conservative therapies in patients with ankle inversion sprain with swelling. 

## 4. Conclusions

Data presented in this case report seem to prove that this intervention was a successful treatment method, but there is no scientific proof that this method will be a successful clinical treatment in all acute ankle inversion sprains with swelling cases. Therefore, a future comparative study is needed which would include the application of various interventions to a large number of patients with acute ankle inversion sprain and swelling. 

## Figures and Tables

**Table 1:** Outcome of the physiological assessment.

Variables	Baseline Score (Right/Left)	Final Score (Right/Left)
Figure-of-eight-20 (cm)	54/57	53/53
Visual analog scale (score)	5/7	0/0
The weight bearing lunge test (cm)	2/0	12/12

**Table 2:** Outcomes of the patient-specific functional scale.

Variables	Baseline Score (Right/Left)	Final Score (Right/Left)
PSFS (score)	17/50	50/50
Activity 1 (Walking downstairs)	4/10	10/10
Activity 2 (Standing on one leg)	3/10	10/10
Activity 3 (Squatting)	4/10	10/10
Activity 4 (Performing ankle circles)	3/10	10/10
Activity 5 (Performing heel lifts)	3/10	10/10

PSFS, patient-specific functional scale.

**Table 3:** Static balance using Zebris and foot ankle outcome score.

Variables	Baseline Score	Final Score
COP path length (mm)	208	103
Foot ankle outcome score (%)	41	97

COP, center of pressure.
